# Captivity and Infection by the Fungal Pathogen *Batrachochytrium salamandrivorans* Perturb the Amphibian Skin Microbiome

**DOI:** 10.3389/fmicb.2019.01834

**Published:** 2019-08-23

**Authors:** Kieran A. Bates, Jennifer M. G. Shelton, Victoria L. Mercier, Kevin P. Hopkins, Xavier A. Harrison, Silviu O. Petrovan, Matthew C. Fisher

**Affiliations:** ^1^Department of Zoology, University of Oxford, Oxford, United Kingdom; ^2^Department of Infectious Disease Epidemiology, MRC Centre for Global Infectious Disease Analysis, School of Public Health, Imperial College London, London, United Kingdom; ^3^Institute of Zoology, Zoological Society of London, London, United Kingdom; ^4^College of Life and Environmental Sciences, University of Exeter, Exeter, United Kingdom; ^5^Department of Zoology, University of Cambridge, Cambridge, United Kingdom; ^6^Froglife, Peterborough, United Kingdom

**Keywords:** *Batrachochytrium salamandrivorans*, chytridiomycosis, microbiome, microbial ecology, amphibian

## Abstract

The emerging fungal pathogen, *Batrachochytrium salamandrivorans* (*Bsal*) is responsible for the catastrophic decline of European salamanders and poses a threat to amphibians globally. The amphibian skin microbiome can influence disease outcome for several host-pathogen systems, yet little is known of its role in *Bsal* infection. In addition, many experimental *in-vivo* amphibian disease studies to date have relied on specimens that have been kept in captivity for long periods without considering the influence of environment on the microbiome and how this may impact the host response to pathogen exposure. We characterized the impact of captivity and exposure to *Bsal* on the skin bacterial and fungal communities of two co-occurring European newt species, the smooth newt, *Lissotriton vulgaris* and the great-crested newt, *Triturus cristatus*. We show that captivity led to significant losses in bacterial and fungal diversity of amphibian skin, which may be indicative of a decline in microbe-mediated protection. We further demonstrate that in both *L. vulgaris* and *T. cristatus, Bsal* infection was associated with changes in the composition of skin bacterial communities with possible negative consequences to host health. Our findings advance current understanding of the role of host-associated microbiota in *Bsal* infection and highlight important considerations for *ex-situ* amphibian conservation programmes.

## 1. Introduction

Microbial communities associated with amphibian skin are increasingly recognized for their ecological complexity and importance in pathogen defense (Bletz et al., 2013; Walke and Belden, 2016). Understanding how amphibian skin microbial communities respond to abiotic and biotic stressors, such as changes in host environment and pathogen attack remains an important question with applications in the fields of microbial ecology and amphibian conservation. Studies to date have demonstrated that skin-associated bacterial community structure may be shaped by the host's environment and is linked to disease outcome in amphibians infected by fungal pathogens such as *Batrachochytrium dendrobatidis* (Lauer et al., 2007; Harris et al., 2009; Jani and Briggs, 2014; Loudon et al., 2014a; Bates et al., 2018). As such, in recent years the recognition of the microbiome's protective role in infection has led to a search for pathogen-inhibiting probiotics and microbial manipulations that could mitigate disease severity and subsequently be utilized as a conservation tool (Bletz et al., 2013; Kueneman et al., 2016).

In 2013, a pathogenic chytrid fungus, *Batrachochytrium salamandrivorans* (*Bsal*), was discovered that was attributed to a 96% decline in the number of fire salamanders (*Salamandra salamandra*) in the Netherlands over just 3 years (Spitzen-van der Sluijs et al., 2013). *Bsal* has since spread to other countries in Europe (Martel et al., 2013, 2014) and threatens amphibians worldwide (Yap et al., 2015). While *Bd* and *Bsal* are closely related phylogenetically and occupy similar niches as the only known species within the chytridiomycota capable of infecting vertebrates (Berger et al., 1998; Martel et al., 2013), they show marked differences in their biology. *Bd* infects over 500 amphibian species (Stuart et al., 2004; Fisher et al., 2009) that span all amphibian orders, whereas *Bsal* is thought to have a narrower host range limited mostly to caudates (Martel et al., 2014). *Bd* and *Bsal* also differ in their pathogenesis with *Bd* causing hyperkeratosis and hyperplasia of the amphibian epidermis, compared to lesions and focal necrosis in *Bsal* (Martel et al., 2013). Prior studies have shown variability in *Bsal* susceptibility between caudate species (Martel et al., 2014), however little is known of the determinants of disease outcome. Given its importance in *Bd* infection, the amphibian skin microbiome is a candidate driver of within- and between-species variability in response to *Bsal* exposure. Although studies have shown that the host skin microbiota is important in *Bd* infection outcome (Jani and Briggs, 2014; Bates et al., 2018), little is known of the *in-vivo* host microbiome response to *Bsal* infection (Bletz et al., 2018). A prior study showed that the host microbiota may be important in *Bsal* infection outcome by demonstrating that inoculation of the skin of fire salamanders with *Bsal* inhibiting bacteria slowed the rate of disease progression (Bletz et al., 2018). Importantly, it is not possible to predict the impact of *Bsal* on amphibian microbiota based on prior *Bd* studies, or to presume a microbiome response similar to that of *Bd*. This is due to a range of factors including intrinsic biological differences between *Bd* and *Bsal* (Farrer et al., 2017) and the variable responses that single bacterial strains can have with different pathogen isolates. For example, it is well-established that the same bacterial strain can either be inhibitory or growth-promoting depending on what *Bd* genotype it is in co-culture with and that inhibition can vary based on microbial community composition (Antwis et al., 2015; Antwis and Harrison, 2018). More recent studies have shown that certain bacteria isolated from amphibian skin inhibit *Bsal*, but not always *Bd in-vitro* (Muletz-Wolz et al., 2017). Further, *Bd* and *Bsal* metabolites can modulate growth of bacteria in different ways (Woodhams et al., 2018). Together these findings suggest that differences are likely to exist in the way that *Bsal* and *Bd* interact with the microbiome of amphibians, reinforcing the importance of investigating the *in-vivo* host microbial response to *Bsal* exposure.

Understanding the biological differences that may exist between wild and captive amphibians represents an important question for several reasons. Firstly, a great deal of our understanding of wildlife diseases comes from laboratory challenge experiments and it is therefore of interest to know how tractable an experimental system may be in terms of a species' wild ecology. Secondly, a common conservation strategy for threatened species is the establishment of captive assurance populations that seek to minimize changes between the wild and captivity so as to enhance the chance of success of future wild reintroductions (Mendelson et al., 2006; Gascon, 2007). Despite recognition of the importance of the microbiome in host health and pathogen defense and the potential for captivity based microbiome bioaugmentation (Bletz et al., 2013), few studies have investigated the impact of captivity on the amphibian skin microbiome (Becker et al., 2014; Loudon et al., 2014b; Bataille et al., 2016; Kueneman et al., 2016; Sabino-Pinto et al., 2016) and none have investigated this with respect to *Bsal* mitigation. Prior studies have yielded mixed results with reductions in bacterial alpha diversity and depletion of chytrid-inhibiting bacteria in captive compared to wild individuals for some host species (Loudon et al., 2014b; Bataille et al., 2016; Kueneman et al., 2016; Sabino-Pinto et al., 2016), while increased alpha diversity was observed in other species (Becker et al., 2014). In addition, studies investigating the impact of captivity on the amphibian skin microbiome have neglected to examine microbial kingdoms other than bacteria, despite recent advances demonstrating that fungi may be equally important to host health and chytrid disease resistance (Kearns et al., 2017). In other systems the host fungal community (also known as the mycobiome) has been shown to be integral to host health. For example, humans with enteric *Clostridium difficile* infection often present dysbiosis of the gut mycobiome with proliferation of *Candida albicans* and reduced fungal diversity (Zuo et al., 2018). Importantly, we cannot predict that both host bacterial and fungal communities will respond in the same way to a change in environment, highlighting the need for a more holistic outlook of the amphibian skin microbiome in terms of the microbial kingdoms investigated. Understanding the effect of captivity on the host microbiome is especially important with regard to *Bsal* since models suggest that field-based interventions are unlikely to be successful owing to disease outbreaks being capable of occurring at lower population densities than host populations exist in the wild, thus making natural pathogen fade out unlikely (Schmidt et al., 2017). In addition, the only currently effective *Bsal* treatments are captivity based (Blooi et al., 2015a,b). Enhancing our empirical understanding of how both the transition from the wild to captivity and *Bsal* infection affects the amphibian skin microbiome is therefore likely to be important in informing future captivity-based conservation interventions.

In this study, we combine field and laboratory studies to investigate how the amphibian skin microbiome changes with the transition from the wild to captivity followed by exposure to *Bsal*. We focus on two UK caudate species, the smooth newt (*Lissotriton vulgaris*) and the great crested newt (*Triturus cristatus*). While *L. vulgaris* is widespread in the UK, *T. cristatus* is more localized in distribution and declining in many parts of its natural range (Edgar et al., 2005). *T. cristatus* is also listed as a protected species in Annexes II and IV of the European Commission Habitats Directive and under the UK Wildlife and Countryside Act 1981. The long-term population viability of *T. cristatus* is thus particularly vulnerable to local disease outbreaks. The susceptibility of captive-raised T*. cristatus* to *Bsal* has been tested in a prior study (Martel et al., 2014) that showed mortality in all infected animals. Meanwhile, no study to date has investigated the lethality of *Bsal* in *L. vulgaris*. Testing the effect of *Bsal* on endemic UK species and the possible risk it poses to wild populations is vital given the recent emergence of *Bsal* in private collections (Cunningham et al., 2015) and the continued spread of pathogenic chytrids through the global trade (O'Hanlon et al., 2018) suggesting a wild outbreak is possible. Understanding the influence of captivity and *Bsal* infection on the amphibian skin microbiome and host health could therefore inform effective captivity based measures that seek to maximize natural microbe-derived pathogen protection.

## 2. Methods

### 2.1. Field Sampling and Captivity Study

A total of 15 adult *Triturus cristatus* and 15 adult *Lissotriton vulgaris* were collected at night from a reserve in Cambridgeshire, UK. Animals were rinsed with sterile water to remove transient bacteria on the skin (Culp et al., 2007) and using a single sterile MW100 rayon tipped dry swab (MWE Medical Wire, Corsham, UK), the skin microbiome was sampled by swabbing the ventral and dorsal surfaces 10 times and the fore- and hindlimbs five times. Swabs were stored at −80°C until processed. Animals were transferred to individual 1.6 L plastic boxes containing moss collected from the field site and transported to the Central Biomedical Services (CBS) Unit at Imperial College London. In captivity animals were housed individually in plastic boxes containing a damp paper towel substrate and a cover object. Enclosures were cleaned with Rely + On Virkon (Antect International Ltd., Suffolk, UK) and animals were fed mealworms (*Tenebrio molitor*) or crickets (*Acheta domesticus*) *ad libitum* twice weekly. The animal room was kept on a 12 h light/dark cycle and was maintained at 16°C. At 2 weeks post-capture animals were swabbed again to measure the effects of captivity on the skin bacterial and fungal community. The “captivity study” encapsulated the time from the initial capture and swabbing in the field, to 2 weeks post-capture in captivity when the second swab was taken.

### 2.2. *Bsal* Exposure Experiment

To compare the response of the two different newt species to *Bsal* exposure, experiments were designed to be as similar as possible to those described in a previous study (Martel et al., 2014). Briefly, *Batrachochytrium salamandrivorans* (isolated from a *Salamandra salamandra* outbreak in the Netherlands, isolate AMFP13/1) was grown in 25cm^3^ Nunc™ tissue culture flasks (Thermo Fisher Scientific, Massachusetts, USA) containing mTGhL liquid media (8 g tryptone, 2 g gelatin hydrosylate, 4 g lactose, 1000 ml distilled water) and incubated at 15°C. At 14 days post-capture (corresponding to the final day of the “captivity study”), ten individuals from each newt species were randomly assigned to a treatment group and exposed to 500 μL of mTGhL media containing 50 × 10^4^ *Bsal* zoospores. The remaining five individuals from each species were assigned to a control group and exposed to 500 μL of mTGhL liquid media. The inoculum was pipetted directly onto the dorsum of the animal. During exposure, *T. cristatus* were placed individually in 0.7 L plastic boxes and *L. vulgaris* were placed in sterile petri dishes for 22 h. Animals were weighed and swabbed prior to *Bsal* infection on day 1 of the experiment and then every 7 days post-infection for a period of 58 days. On day 58 of the experiment surviving animals were euthanized by an overdose of tricaine methanesulfonate (MS222) and subsequent destruction of the brain following UK Home Office animal procedure guidelines. In agreement with ethical protocols, any animals exhibiting pre-defined endpoint criteria (lack of righting reflex within 5 s of being inverted, persistent skin lesions covering over 20% of the body or that became septic, >20% loss in body weight) were euthanized prior to day 58. An overview of the design for the wild/captive and *Bsal* study is included in Figure S1.

### 2.3. Sample Processing, DNA Extraction, and Quantification of *Bsal* Infection

Genomic DNA was extracted from swabs using a bead beating protocol (Boyle et al., 2004) and diluted 1/10 before undergoing subsequent PCR based analyses. Quantification of *Bsal* infection load was done using qPCR amplification following a modified published method (Boyle et al., 2004) that included a *Bsal* specific probe (STerCVIC), forward primer (STerFC), and reverse primer (STerT) (Blooi et al., 2013). Each sample was run in duplicate and with *Bsal* standards of 100, 10, and 1 genomic equivalents (GE). A distilled water negative control was also included. Samples were considered positive if both wells gave a GE of >0.1.

### 2.4. Bacterial Microbiome Sample Processing

DNA extracted from swabs was used to amplify the V4 region of the 16S rRNA gene using custom barcoded primers and PCR conditions adapted from a prior study (Kozich et al., 2013). PCR conditions consisted of a denaturing step of 95°C for 15 min, followed by 28 cycles of 95°C for 20 s, 50°C for 60 s, 72°C for 60 s, and a final extension step of 72°C for 10 min. Each PCR including a negative water control was performed in triplicate. Amplicons were visualized on a 2% agarose gel and pooled yielding a final per sample volume of 24 μL. Pooled amplicon DNA was purified using an Ampure XP PCR purification kit (Beckman Coulter, California, USA). Following purification, 1 μL of each combined sample was pooled into a preliminary library and the concentration was determined using Qubit fluorometric quantification (Life Technologies, California, USA). Amplicon quality and incidence of primer dimer was assessed using an Agilent 2200 TapeStation system (Agilent Technologies, California, USA). A titration run of 300 sequencing cycles was performed on a MiSeq instrument (Illumina, California, USA) to quantify the number of reads yielded per sample from the preliminary library. An equimolar concentration of each sample was then pooled into a final composite library based on the index representation from the titration run and subsequently sequenced on a 500 cycle MiSeq run with a 250 bp paired-end strategy using V2 chemistry.

### 2.5. Fungal Mycobiome Sample Processing

DNA extracted from swabs was used to amplify the ITS2 region of the fungal internal transcribed spacer (ITS) using custom barcoded primers (Kozich et al., 2013) and the following PCR conditions: denaturing step of 95°C for 2 min, followed by 35 cycles of 95°C for 20 s, 50°C for 20 s, 72°C for 5 min and a final extension step of 72°C for 5 min. Each PCR plate included a negative control and was performed in duplicate. Amplicons were visualized on a 1.5% agarose gel and pooled yielding a final per sample volume of 50 μL. Pooled amplicon DNA was purified using AMPure XP bead clean-up (Beckman Coulter, California, USA). Qubit fluorometric quantification (Life Technologies, California, USA) was used to determine the concentration of each purified sample, which were equimolar pooled to create the final library sample. This pooled sample was run on an Agilent 2200 TapeStation system (Agilent Technologies, California, USA) to assess amplicon distribution and presence of primer dimer. The sample underwent 300 bp paired-end sequencing using V3 chemistry on an Illumina MiSeq platform.

### 2.6. Bacterial Microbiome Analysis

Sequences were processed using MOTHUR (Schloss et al., 2009) following a previously described method (Kozich et al., 2013). Paired-end reads were split by sample and assembled into contigs. Sequences were quality filtered by removing ambiguous base calls, removing homopolymer regions longer than 8 bp, and trimming reads longer than 275 bp. Duplicate sequences were merged and aligned with 16S reference sequences from the SILVA small-subunit rRNA sequence database (Pruesse et al., 2007). A pre-clustering step grouped sequences differing by a maximum of 2 bp. Chimeric sequences were removed using UCHIME (Edgar et al., 2011) as implemented in MOTHUR. 16S rRNA gene sequences were clustered into groups according to their taxonomy at the level of order and assigned operational taxonomic units (OTUs) at a 3% dissimilarity level. Sequences were taxonomically classified with an 80% bootstrap confidence threshold using a naive Bayesian classifier with a training set (version 9) made available through the Ribosomal Database Project (http://rdp.cme.msu.edu) (Wang et al., 2007). Sequences derived from chloroplasts, mitochondria, archaea, eukaryotes, or unknown reads were eliminated. The number of sequences per sample ranged from 17,804 to 63,367. To mitigate the effects of uneven sampling (Schloss et al., 2011) all samples were rarefied to 17,804 sequences corresponding to the size of the lowest read sample. Any contaminant sequences identified from negative controls were removed using the decontam package (Davis et al., 2018). OTUs making up <0.01% of the total reads were removed (Bokulich et al., 2013). The proportion of taxa at each taxonomic level designated as “unclassified” are shown in Table S1. Downstream analysis of OTUs was carried out using the package Phyloseq (McMurdie and Holmes, 2013) in R version 3.4.1 (R Core Team, 2017).

### 2.7. Fungal Mycobiome Analysis

Analysis of fungal communities was performed for the captivity study only. Following sequencing, forward and reverse reads were assigned to samples according to dual index combinations and were paired using Paired-End reAd mergeR (PEAR) (Zhang et al., 2014). Paired-end reads were trimmed by per-base quality score using MOTHUR (Schloss et al., 2009) and reads shorter than 50 bp or containing ambiguous base calls were removed. UCHIME (Edgar et al., 2011) was used to identify and remove chimeric sequences, and remaining sequences were clustered into Operational Taxonomic Units (OTUs) based on 97% similarity using Cd-hit (Li and Godzik, 2006). The most abundant sequence in each OTU was used for BLASTn searches against the User-friendly Nordic ITS Ectomycorrhiza (UNITE) database (Koljalg et al., 2005). Unidentified sequences or those belonging to kingdoms other than “fungi” were removed, as were fungal sequences with BLASTn search result e-values >e20 or identity <85%. Fungal sequences were recovered from 32 samples in total. The number of sequences per sample ranged from 579 to 38,919. To mitigate the effects of uneven sampling (Schloss et al., 2011) all samples were rarefied to 1,155 sequences resulting in two samples being discarded. The proportion of taxa at each taxonomic level designated as “unclassified” in the rarefied data set are shown in Table S1. OTUs making up <0.01% of the total reads were removed (Bokulich et al., 2013). Downstream analysis of OTUs was carried out using the package Phyloseq (McMurdie and Holmes, 2013) in R version 3.4.1 (R Core Team, 2017).

### 2.8. Statistical Analysis

#### 2.8.1. Testing the Effects of Captivity on Skin Fungal and Bacterial Communities

To test the effect of captivity on the newt skin bacterial and fungal communities, we calculated the observed species richness, Shannon diversity and beta diversity based on the Bray-Curtis dissimilarity matrix using the phyloseq package (McMurdie and Holmes, 2013) in R version 3.4.1 (R Core Team, 2017). To test how bacterial and fungal species richness and Shannon diversity differed between capture and after 2 weeks in captivity, we carried out a Wilcoxon test for each newt species. Beta diversity was visualized using detrended correspondence analysis (DCA) and the effects of captivity, host species and their interaction on skin fungal and bacterial communities were statistically tested using permutational multivariate analysis of variance (PERMANOVA) (Anderson, 2001) using the adonis function in the vegan package (Oksanen et al., 2016). To assess bacterial and fungal OTUs that differed in abundance in the wild vs. captivity for each newt species, we used indicator analysis (Dufrene and Legendre, 1997) using the labdsv package (Roberts, 2016). An indicator score of >0.7 and adjusted *p*-value <0.05 was used as a cut-off to define informative OTUs (Becker et al., 2015; Longo and Zamudio, 2017).

#### 2.8.2. Testing the Effects of *Bsal* Infection on Skin Bacterial Communities

To determine the effect of *Bsal* exposure on the microbiome, we calculated both species richness and beta diversity based on Bray-Curtis dissimilarity using the phyloseq package (McMurdie and Holmes, 2013) in R version 3.4.1 (R Core Team, 2017). To examine the effect of *Bsal* exposure and infection on bacterial species richness, mixed linear models were used for each newt species with the general formula Richness ~ Day + Treatment + Mass + Day * Mass + Treatment * Mass + Day * Treatment + log10(GE + 1) + (1|Individual_ID) using the lme4 package (Bates et al., 2015). *P*-values were approximated using the Kenward-Roger method using the afex package (Singmann et al., 2017). Beta diversity was visualized using detrended correspondence analysis (DCA) plots and differences in beta diversity based on treatment group and *Bsal* infection status were investigated using PERMANOVA for days 1, 28, and 56 of the experiment. To test if the microbiome of animals pre-*Bsal* exposure was linked to infection outcome, we tested for a correlation between the bacterial Bray-Curtis distance matrix prior to infection at day 1 and the *Bsal* distance matrix at 28 and 56 days post-*Bsal* exposure using Mantel tests. For the Mantel tests, *Bsal* infection data was converted to pairwise Euclidean distances. Correlations between *Bsal* and bacterial community distance matrices were performed for animals in the *Bsal* exposed groups only, since we were interested in associations between bacterial communities and infection severity. To test if *Bsal* infection load affected host bacterial communities, we tested for a correlation between the *Bsal* infection load matrix and the bacterial distance matrix at day 28 for both host species and at day 56 for *T. cristatus* (all *L. vulgaris* had cleared infection by day 56). To test for differences in the abundance of core microbiome OTUs (with a relative abundance >1%), we performed indicator analysis for each host species with *Bsal* infection status (control, exposed *Bsal* negative, exposed *Bsal* positive) used as predictor variables at days 1, 28, and 56. Differences in survival among animals in the *Bsal* exposure experiment was investigated using a cox proportional-hazard regression model in the survival package (Therneau, 2015) with mass at the beginning of the experiment, species, and GE at time of death or at the end of the experiment included as co-variates.

### 2.9. Ethics Statement

Animal work was conducted under UK Home Office Project License PPL 70/8402 held by Matthew Fisher and was reviewed by the Imperial College London Animal Welfare Ethical Review Board for approval. The experiment was carried out in accordance with The Animals (Scientific Procedures) Act of 1986 Directive 2010/63/EU and followed all of the Codes of Practice which reinforce this law, including all elements of housing, care and euthanasia. Animals were collected in the wild under Natural England License 2015-15771-SCI-SCI and following ethical review by the board of Froglife.

## 3. Results

### 3.1. Captivity Impacts Bacterial and Fungal Diversity and Community Structure

**Figure 1:**
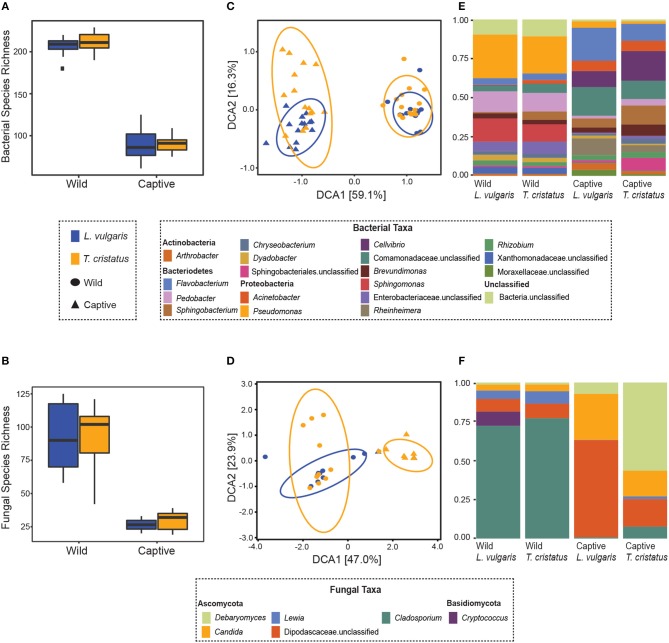
Microbiome of animals in the wild and after 14 days in captivity. Boxplots displaying observed species richness in wild and captive *Lissotriton vulgaris* and *Triturus cristatus* for **(A)** bacteria **(B)** fungi. Boxes represent 25th and 75th percentile, the horizontal line is the median, whiskers are maximum and minimum values. Detrended correspondence analysis (DCA) plots of beta diversity in wild and captive *L. vulgaris* and *T. cristatus* for **(C)** bacteria **(D)** fungi. Ellipses indicate 95% confidence intervals. Where ellipses are absent, insufficient samples were present. Stacked bar plots of **(E)** bacterial taxa with relative abundance >1% and **(F)** fungal taxa with relative abundance >1%. Sample sizes bacteria: *n* = 15 (wild *L. vulgaris*), *n* = 15 (wild *T. cristatus*), *n* = 15 (captive *L. vulgaris*), *n* = 15 (captive *T. cristatus*). Sample sizes fungi: *n* = 10 (wild *L. vulgaris*), *n* = 11 (wild *T. cristatus*), *n* = 2 (captive *L. vulgaris*), *n* = 7 (captive *T. cristatus*).

**Figure 2:**
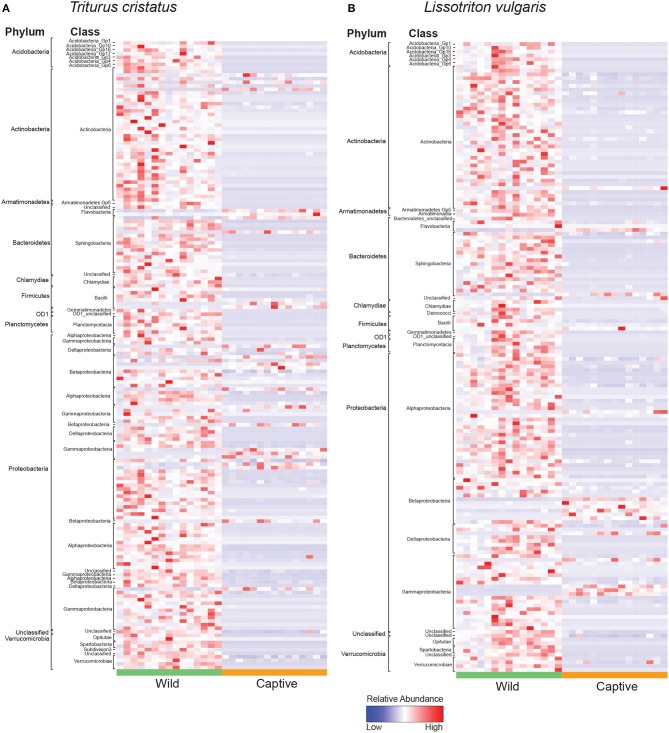
Heatmap of normalized relative abundance of bacterial indicator OTUs for animals in the wild and after 14 days in captivity labeled by phylum and class for **(A)** *T. cristatus* **(B)** *L. vulgaris*. Sample sizes: *n* = 15 (wild *L. vulgaris*), *n* = 15 (wild *T. cristatus*), *n* = 15 (captive *L. vulgaris*), *n* = 15 (captive *T. cristatus*).

We show significant reductions in bacterial and fungal species richness associated with the transition from the wild to captivity (measured at initial capture and 2 weeks post-capture) for both host species (bacteria: *T. cristatus* and *L. vulgaris p* < 0.0001, fungi: *T. cristatus* and *L. vulgaris p* < 0.0001, Figures 1A,B). A reduction in bacterial and fungal Shannon diversity was also evident when animals were brought into captivity for both host species (*p* < 0.01, Figure S2). Bacterial and fungal beta diversity differed in wild vs. captive conditions [PERMANOVA, bacteria wild-captive: Pseudo-*F*_(1, 56)_ = 66.902, *R*^2^ = 0.51, *p* = 0.001, fungal wild-captive: Pseudo-*F*_(1, 26)_ = 12.17, *R*^2^ = 0.31, *p* = 0.001] and a host species effect was present for bacterial communities [PERMANOVA, host species: Pseudo-*F*_(1, 56)_ = 5.441, *R*^2^ = 0.041, *p* = 0.006, host species*wild-captive: Pseudo-*F*_(1, 56)_ = 3.947, *R*^2^ = 0.030, *p* = 0.01, Figures 1C,D]. Changes in alpha and beta diversity associated with captivity were mirrored for bacteria and fungi demonstrating a common response across microbial kingdoms. Captivity was associated with compositional changes in the core microbiome of both species with reductions in taxa, such as *Cladosporium* and *Pseudomonas* (Figures 1E,F). Indicator analysis of the microbiome of animals in the wild vs. captivity identified 176 and 166 differentially abundant bacterial OTUs in *T. cristatus* and *L. vulgaris*, respectively (Figures 2A,B; Datas S1, S2). No significant differences in fungal indicator OTU abundance were associated with captivity for *L. vulgaris*, while seven indicator OTUs were identified for *T. cristatus* (Table S2, Figure S3). Both host species demonstrated similar indicator OTU profiles of major bacterial groups (Figures 2A,B) with changes in abundance of a large number of Proteobacteria and Actinobacteria.

### 3.2. *Bsal* Infection Causes Mortality in *L. vulgaris* and *T. cristatus*

**Figure 3:**
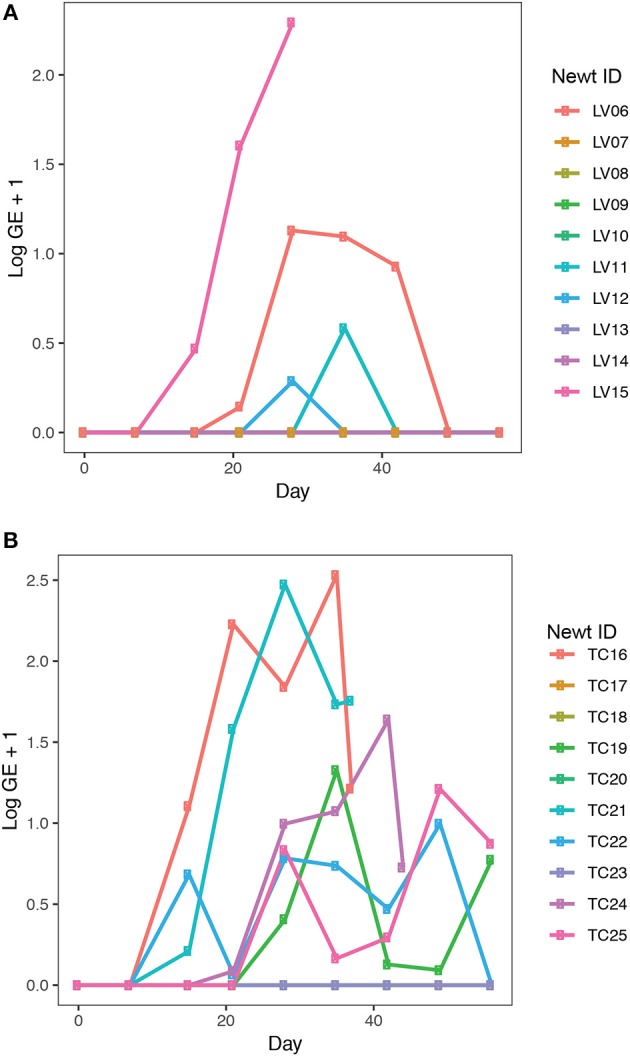
*Bsal* infection intensity in **(A)** *L. vulgaris*, **(B)** *T. cristatus*. Sample sizes: *n* = 10 *Bsal* exposed *T. cristatus, n* = 10 *Bsal* exposed *L. vulagris*.

*Bsal* exposure resulted in infection in 40% (4 out of 10 animals) of *L. vulgaris* and 60% (6 out of 10 animals) of *T. cristatus*. Prevalence and infection intensity fluctuated throughout the experiment for both species (Figures 3A,B, Table S3) with *T. cristatus* exhibiting consistently higher infection intensity and prevalence than *L. vulgaris*. Lesions were evident in 50% of *T. cristatus* and 75% of *L. vulgaris* that tested *Bsal* positive (Table S3). Of the animals that tested positive for *Bsal*, 50% (3 out of 6 infected animals) of *T. cristatus* died, while 25% (1 out of 4 infected animals) of *L. vulgaris* died over the 58 days of the experiment. Of the four *L. vulgaris* that became infected with *Bsal*, three animals cleared infection, while for *T. cristatus* only one of six *Bsal* positive animals cleared infection. Survival analysis showed that infection intensity but not species or mass were significantly associated with mortality (hazard-ratio = 1.07, *p* = 0.022, Table S4).

### 3.3. *Bsal* Infection Alters Host Bacterial Community Structure

**Figure 4:**
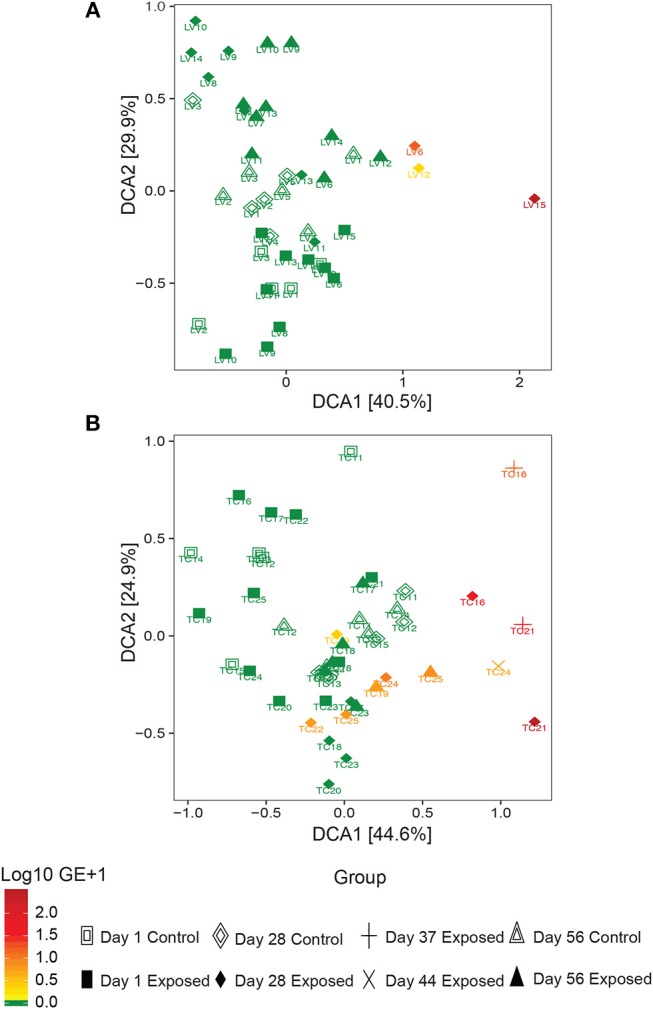
Detrended correspondence analysis of beta diversity in the *Bsal* exposure experiment in **(A)** *L. vulgaris*, **(B)** *T. cristatus*. Each point is labeled with the individuals identity, shape is based on treatment group and day of sampling and color represents *Bsal* infection intensity (log10 GE+1). Initial sample sizes at day 1: *n* = 10 *Bsal* exposed *T. cristatus, n* = 10 *Bsal* exposed *L. vulagris, n* = 5 control *T. cristatus, n* = 5 control *L. vulgaris*.

**Figure 5:**
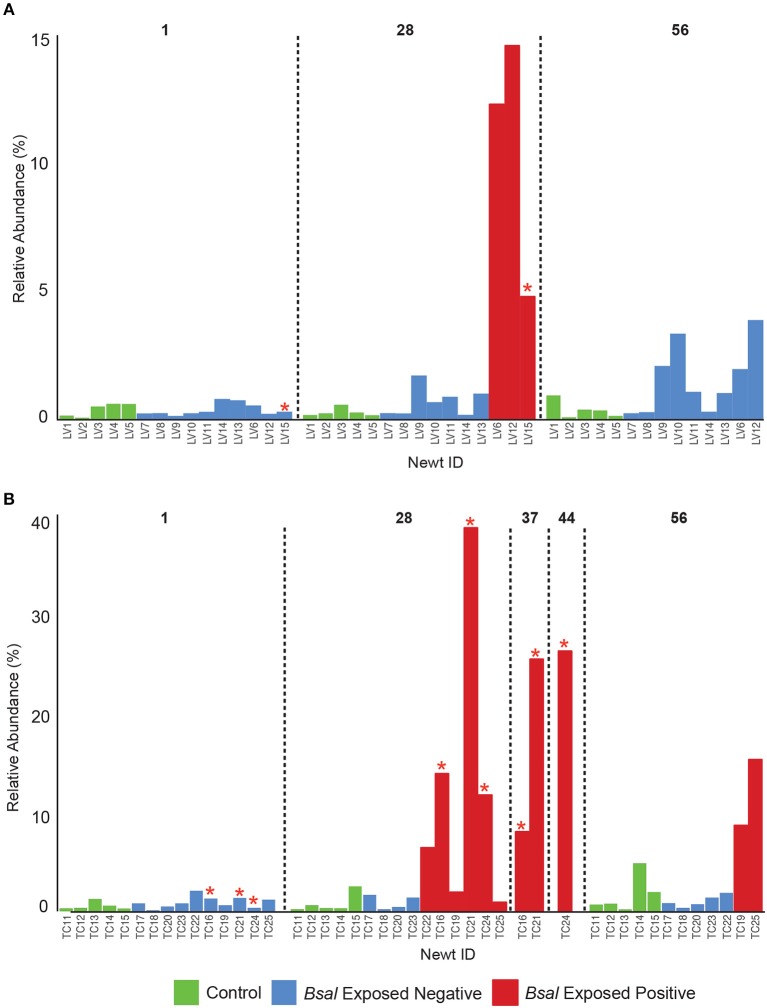
Relative abundance of *Stenotrophomonas* on the skin of individual newts for **(A)** *L. vulgaris*, **(B)** *T. cristatus*. Red stars indicate animals that died of *Bsal* infection. Initial sample size at day 1: *n* = 10 *Bsal* exposed *T. cristatus, n* = 10 *Bsal* exposed *L. vulagris, n* = 5 control *T. cristatus, n* = 5 control *L. vulgaris*.

Observed bacterial species richness did not differ significantly for either host species based on day of sampling, *Bsal* exposure, host mass (and their pairwise interactions) or *Bsal* infection intensity (*p* > 0.05). Beta diversity differed only on day 28 of the infection experiment for both host species based on disease status (*Bsal* positive or negative) (PERMANOVA *T. cristatus* Pseudo-*F*_(1, 12)_ = 3.39, *R*^2^ = 0.196, *p* = 0.011, *L. vulgaris* Pseudo-*F*_(1, 12)_ = 4.46 *R*^2^ = 0.249, *p* = 0.002, Figures 4A,B) but not based on treatment group. In *L. vulgaris*, on day 28 two infected individuals demonstrated microbiome perturbation, based on a shift in beta diversity that differed from healthy controls, but cleared infection and returned to a microbiome state that did not differ significantly from control animals by day 56 (Figure 4A). Bacterial communities prior to *Bsal* exposure were not correlated with infection intensity at 28 and 56 days (*T. cristatus* only) post-exposure for both host species (Mantel test *p* > 0.05). Differences in bacterial community structure of *Bsal* exposed animals was however correlated with infection load measured on the same day for day 28 (*L. vulgaris* Day 28 Mantel test: *p* = 0.003, Spearman's correlation coefficient = 0.70; *T. cristatus* Day 28 Mantel test: *p* = 0.001, Spearman's correlation coefficient = 0.85, Day 56 Mantel test: *p* > 0.05). Indicator analysis of microbiome samples based on control, exposed *Bsal* negative and/or exposed *Bsal* positive animals revealed significant differences in taxa abundance on day 28 for both host species and also on day 56 for *L. vulgaris*. No significant differences in taxa abundance were found on day 1 for both host species. A total of four and one differentially abundant bacterial operational taxonomic units (OTUs) were identified in *L. vulgaris* and *T. cristatus*, respectively on day 28 (Table S5, Figure S4). Both *L. vulgaris* and *T. cristatus* exhibited an indicator taxa identified as *Stenotrophomonas* (Figures 5A,B), which was strongly associated with *Bsal* infection in both species (indval > 0.8, adjusted *p*-value < 0.05). In *L. vulgaris, Chryseobacterium* was identified as an indicator taxa for *Bsal* exposed positive animals on day 28 and *Bsal* exposed negative animals on day 56. This result is due to the relatively high abundance of *Chryseobacterium* that remained on animals that had cleared infection at day 56 relative to control animals (Figure S4).

## 4. Discussion

### 4.1. Summary

The amphibian skin microbiome is an important first line of defense against pathogens. Changes in environment may alter the host microbiota with potential consequences to host-microbe-pathogen interactions and host health. In this study, we investigated the importance of host environment on the microbiome by sampling dermal bacterial and fungal communities of *L. vulgaris* and *T. cristatus* in the wild before relocating them to captivity and measuring changes in microbial composition and diversity at 2 weeks post-capture. We then exposed animals to the fungal pathogen *Batrachochytrium salamandrivorans* (*Bsal*) or a sham control infection and monitored changes in host bacterial communities and host survival over a 58 day period. Our results demonstrate that captivity and *Bsal* infection impact the skin microbial communities of both host species. We demonstrate a significant decline in bacterial and fungal species diversity when animals are brought into captivity in addition to changes in microbial community composition that may have implications to microbe mediated immunity. While we found no evidence that host bacterial community structure pre-infection correlated with disease outcome, we did find that *Bsal* infection load correlated with bacterial community composition and that bacterial beta diversity significantly differed in *Bsal* infected vs. uninfected animals. These findings indicate that *Bsal* infection is associated with perturbations of the host microbiota and that pathogen driven changes in host microbial ecology may bias negative health outcomes.

### 4.2. Captivity Diminishes Fungal and Bacterial Diversity

We demonstrate that captivity results in a decrease in bacterial and fungal species richness and changes in beta diversity. These results support the findings of previous studies (Becker et al., 2014; Kueneman et al., 2016) and are likely correlated with a reduction in environmental microbial diversity that reduce the number of possible microbial species that can colonize a host (Harrison et al., 2017). In addition, changes to the host environment may alter the selective pressures on the existing hosts microbiota (Harrison et al., 2017). Of note in this study was the similar change in alpha and beta diversity in both bacteria and fungi when animals were brought from the wild to captivity suggesting that these microbial kingdoms respond in broadly similar ways to the selection pressures associated with the amphibian skin environment.

The reduction in microbial Shannon diversity and shift in beta diversity that coincide with the transition from the wild to captivity may also change the ecological dynamics of the skin microbiota, with potential consequences to host health. For example, it may be hypothesized that the risk of infection is reduced in a low microbial diversity captive environment with fewer potential pathogen reservoirs. This hypothesis is partially supported by our analysis of differentially abundant bacterial OTUs for which we show a decline in captivity of the Chlamydiales (Figures 2A,B) that have previously been associated with amphibian epizootics (Reed et al., 2000). Conversely, captivity may have a negative effect on the host microbiome. Specifically, the captivity associated decline in microbial alpha diversity may reduce the ecological resistance of the host microbiota (Piovia-Scott et al., 2017) rendering individuals more susceptible to pathogen invasion (Piovia-Scott et al., 2017). This is supported by increases in captivity in the relative abundance of the amphibian fungal pathogen *Basidiobolus ranarum* in *T. cristatus* (Figure S3). *Basidiobolus ranarum* has been shown to be a causative agent of population declines in the wild and causes fatal mycotic dermatis and skin sloughing in infected animals (Taylor et al., 1999a,b). The increased abundance of *B. ranarum* in captivity may therefore have had an compromising effect on host health that increased infection risk from subsequent *Bsal* exposure. Further evidence for a decline in microbe-mediated protection of the host in captivity comes from the reduction of putatively beneficial bacterial groups, such as the Actinomycetales (Figures 2A,B), which are documented for being prolific producers of antimicrobial compounds (Berdy, 2005) and have previously been associated with resistance to chytrid infection and enzootic disease dynamics in the wild (Bates et al., 2018). In addition, taxa belonging to the genus *Lysobacter* that have previously been associated with inhibition of other pathogenic chytrids (Brucker et al., 2008) also showed a reduction in abundance in captivity.

While we show that captivity is associated with wholesale changes to the host-microbiota, the relevance of such changes to host health remains to be resolved. Ultimately, determining if captivity is linked to changes in microbe-mediated protection and host health requires comparative functional assays of the host skin microbiota in the wild and captivity, in addition to measurements of key host physiological parameters, such as immune function. Importantly, this is the first study of its kind to measure the effect of captivity on fungal communities, which despite being overlooked relative to bacteria, have been shown to confer higher rates of chytrid inhibition in some cases (Kearns et al., 2017). While our analysis of fungal communities was limited by the relatively low recovery of sequences from our samples (particularly for captive *L. vulgaris*), our demonstration of shifts in fungal community diversity when animals transitioned from the wild to captivity underscores the need for future studies to utilize a more holistic view of the microbiome to include kingdoms other than bacteria. Overall, our results demonstrate that host microbial ecology should carefully be considered when transferring animals from the wild to captivity to reduce possible microbiome disturbance, prevent proliferation of opportunistic pathogens and preserve microbes that are beneficial to host health. For *ex-situ* conservation programmes that aim to rescue or re-establish wild amphibian populations using captive-bred stock, the possible implications are complex and may suggest that maintenance or “rewilding” of the skin microbiome would be an essential aspect underpinning successful re-introductions. In addition, the increased abundance of potential pathogens in captivity underscores the importance of rigorous biosecurity measures to minimize the risk of introducing disease agents into novel host environments through re-introduction programmes.

### 4.3. Host Species Responses to *Bsal* Exposure

*Bsal* infection resulted in mortality in both *L. vulgaris* and *T. cristatus*. While mortality was higher in *T. cristatus*, this was not statistically significant when compared to *L. vulgaris*. The range of intensity of infection was found to be higher in *T. cristatus*, which had a maximum GE of 338 compared to a maximum GE of 195 for *L. vulgaris*. These data suggest that *T. cristatus* may be more susceptible to *Bsal* infection than *L. vulgaris*, however a follow-up experiment with a larger sample size, ideally with animals from multiple populations is required to more robustly test this. Our findings counter those of a previous study that found 100% mortality in *T. cristatus* and a species effect on survival (Martel et al., 2014). The increased survival of *T. cristatus* in this study compared to the prior study (Martel et al., 2014) may be explained by a variety of factors. In particular, while our study used the same *Bsal* isolate as the prior study and thus we expect pathogen differences due to laboratory conditions to be minimal, the animals used in both studies differed in their provenance. Specifically, this study used animals collected from the UK (vs. the Netherlands) and there may well be differences in host response to infection driven by factors such as host genetics, immunity and microbiome composition. These findings highlight the importance of investigating a geographically diverse range of hosts from the same species when conducting species level risks assessments of emerging pathogens. While this additional confirmation of *Bsal* induced death in *T. cristatus* warrants serious concern, the higher survivorship shown here may suggest a less catastrophic outlook in the event of a wild *Bsal* outbreak for this species than the total mortality previously predicted (Martel et al., 2014).

### 4.4. *Bsal* Infection Disrupts Host Skin Bacterial Communities

While the bacterial communities of animals from both host species pre-*Bsal* exposure did not correlate with *Bsal* infection, bacterial community structure did differ significantly in infected vs. uninfected individuals. These findings are consistent with results of prior studies on *Bd* (Jani and Briggs, 2014) and suggest that host skin bacterial communities may not be a predictor of disease resistance, but rather that *Bsal* infection is associated with community level microbiome disruption and subsequent negative health effects (Zaneveld et al., 2017). Surprisingly, two *L. vulgaris* (LV6 and LV12) and one *T. cristatus* (TC22) tested positive for *Bsal* and demonstrated evidence of microbiome perturbation on day 28 of the experiment, but subsequently cleared infection by day 56. In both cases *Bsal* clearance was associated with a change in microbiome profile with the subsequent community structure returning to a similar composition and beta diversity to that of control animals. In both host species, animals that cleared infection displayed less distinct changes in beta diversity relative to controls than animals that succumbed to *Bsal* induced mortality. This suggests that the extent of bacterial microbiome perturbation is associated with infection severity and that animals that eventually die may also experience prolonged microbial disruption and possible dysbiosis. Investigating the mechanisms underpinning infection clearance, whether driven by host factors such as secretion of antimicrobial peptides, or pathogen inhibition by microbes poses an important question in our understanding of *Bsal* disease dynamics.

Indicator analysis revealed that bacterial taxa associated with disease state differed between host species with the exception of one OTU classified as *Stenotrophomonas*. Strains of *Stenotrophomonas* have been shown to exhibit a wide range of interactions with amphibian-infecting chytrids, with some studies showing an antagonistic relationship (chytrid inhibition) while others have demonstrated synergy (promotion of chytrid growth) (Becker et al., 2015; Muletz-Wolz et al., 2017; Antwis and Harrison, 2018; Bletz et al., 2018). One particular study identified an isolate of *Stenotrophomonas* as inhibitory to *Bd in-vitro*, however when applied as a probiotic on an amphibian host exposed to *Bd in-vivo*, resulted in higher mortality than in animals that were exposed to only *Bd* (Becker et al., 2015). More recently an *in-vivo* experiment in which fire salamanders (*Salamandra salamandra*) were exposed to *Bsal* identified an OTU belonging to the *Stenotrophomonas* genus as significantly increased in abundance in exposed compared to control animals (Bletz et al., 2018). Based on the high abundance of *Stenotrophomonas* on *Bsal* infected animals in this study and in light of findings from prior studies, it would appear that *Stenotrophomonas* in this case is either synergistic with *Bsal* growth, or opportunistically infecting moribund animals. Importantly, *Stenotrophomonas* has been associated with opportunistic infection in other host systems (Adegoke et al., 2017) and in our study only proliferated in abundance once animals became infected with *Bsal*. Co-infection may also be more easily facilitated in *Bsal* infected animals where lesions lack the physical barrier of the skin, thus providing an entry point to the hosts systemic circulation. Interestingly, *Stenotrophomonas* was also identified as an indicator taxa for captive animals in both host species, suggesting that captive conditions favor its proliferation. Evidence of co-infection is further supported by the increased abundance of *Chryseobacterium* in *Bsal* infected *L. vulgaris*. In particular, *Chryseobacterium* has also previously been discovered to proliferate on *Bsal* exposed *Salamandra salamandra* where it was further linked to septacemic pathology (Bletz et al., 2018).

While this study represents an important step in our understanding of how host environment and exposure to *Bsal* impacts the amphibian skin microbiome and host survival, it was limited by the relatively small sample size of animals used (restricted due to the protected status of *T. cristatus*), combined with the fact that not all *Bsal* exposed animals became infected. These factors consequently limit our statistical power in determining the effect of infection on the microbiome and disease dynamics. Finally, although we have identified some convincing microbial associations with *Bsal* infection, we cannot conclusively comment on what functional impact specific taxa have on the host and its microbiota. Microbial culture and *in-vitro* bacteria-*Bsal* competition assays in addition to further animal experiments will yield valuable information regarding the protective or detrimental nature of candidate bacteria on their host. In addition, future studies would benefit from the use of other “omics” methods, such as shotgun metagenomics and metabolomics that provide information on the functional repertoire of the skin microbiota.

Ultimately, our results build on the work of prior studies by demonstrating that captivity results in reduced diversity and changes in species composition of the amphibian skin microbiome which may potentially bias negative health outcomes. In addition, we provide vital insight into the progression of *Bsal* infection by demonstrating a close link between disease outcome and bacterial community structure. Overall, our findings demonstrate that it is prudent for host microbial ecology to be considered in future *Bsal* studies and captivity based conservation programmes.

## Data Availability

Sequence data have been deposited on the BioProject database under accession code PRJNA430498. All other data are available upon request from the authors.

## Author Contributions

KB and VM conducted field surveys and animal experiments. KB, VM, KH, XH, and JS performed data processing and analysis. KB prepared the figures. KB, MF, and SP wrote the manuscript.

### Conflict of Interest Statement

The authors declare that the research was conducted in the absence of any commercial or financial relationships that could be construed as a potential conflict of interest.
