# Supplementary material for: Captivity and Infection by the Fungal Pathogen Batrachochytrium salamandrivorans Perturb the Amphibian Skin Microbiome
Source: Front Microbiol. 2019 Aug 23;10:1834. doi: 10.3389/fmicb.2019.01834 (PMC6716147; doi:10.3389/fmicb.2019.01834)
Supplement: Supplementary file 1 [file Data_Sheet_1.pdf]

Supplementary Material

1 SUPPLEMENTARY DATA

Data S1. Wild-captive indicator analysis for *T. cristatus*.
Data S2. Wild-captive indicator analysis for *L. vulgaris*.

2 SUPPLEMENTARY TABLES AND FIGURES

2.1 Tables

	Kindgom	Phylum	Class	Order	Family	Genus
% Bacterial OTUs Assigned	100	99.86	98.83	97.36	94.13	78.27
% Fungal OTUs Assigned	100	68.24	63.34	60.79	53.34	46.28

Table S1. Percent of total OTUs assigned to each taxonomic level for the rarefied dataset

**Species**	**Group**	**OTU**	**Taxonomy**	**Indicator value**	**Adjusted *p*-value**
T. cristatus	Captive	Otu00370	*Basidiobolus ranarum*	1.0	0.03
T. cristatus	Captive	Otu00023	Unclassified	0.98	0.03
T. cristatus	Captive	Otu00009	*Debaryomyces* sp.	0.98	0.04
T. cristatus	Captive	Otu00001	*Debaryomyces hansenii* sp.	0.96	0.04
T. cristatus	Captive	Otu00578	Unclassified	0.84	0.04
T. cristatus	Wild	Otu00011	*Cladosporium allicinum*	0.95	0.04
T. cristatus	Wild	Otu00164	*Cladosporium* sp.	0.91	0.04

Table S2. Fungal indicator OTUs for *T. cristatus* for wild and captivity.

Species	Day 1	Day 7	Day 15	Day 21	Day 28	Day 35	Day 42	Day 49	Day 56
L. vulgaris prevalence (%)	0.0 (0.0, 30.8)	0.0 (0.0, 30.8)	10.0 (0.25, 44.5)	20.0 (2.5, 55.6)	30.0 (6.7, 65.2)	22.2 (2.8, 60.0)	11.1 (0.28, 48.2)	0.0 (0.0, 33.6)	0.0 (0.0, 33.6)
L. vulgaris lesions (%)	0.0	0.0	10.0	30.0	30.0	22.2	22.2	22.2	0.0
T. cristatus prevalence (%)	0.0 (0.0, 30.8)	0.0 (0.0, 30.8)	30.0 (6.7, 65.2)	40 (12.2, 73.8)	60.0 (26.2, 87.8)	60.0 (26.2, 87.8)	50.0 (15.7, 84.3)	42.9 (9.9, 81.6)	28.6 (3.66, 71.0)
T. cristatus lesions (%)	0.0	0.0	0.0	0.0	0.0	20.0	12.5	14.3	14.3

Table S3. Mean *Bsal* prevalence (95% CI) and proportion of animals with lesions (%) for *Lissotriton vulgaris* and *Triturus cristatus*

**Variable**	**Co-efficient**	**HR (95% CI)**	***p*-value**
Species	2.74	15.540 (3.96e-5 - 6.10e+6)	0.68
Infection intensity (GE)	0.06	1.067 (1.01 - 1.13)	0.02
Start weight	0.32	1.371 (0.52 - 3.63)	0.53

Table S4. Results of survival analysis

Whole model r^2 = 0.516 p = 0.02

**Species**	**Group**	**OTU**	**Taxonomy**	**Indicator value**	**Adjusted *p*-value**
L. vulgaris	Day 28 Control	OTU012	Bacteroidetes	0.98	0.009
L. vulgaris	Day 28 Control	OTU041	Rhodobacteraceae	0.73	0.04
L. vulgaris	Day 28 *Bsal* Positive	OTU019	*Chryseobacterium*	0.96	0.03
L. vulgaris	Day 28 *Bsal* Positive	OTU016	*Stenotrophomonas*	0.92	0.009
T. cristatus	Day 28 *Bsal* Positive	OTU016	*Stenotrophomonas*	0.88	0.04
L. vulgaris	Day 56 *Bsal* Negative	OTU019	*Chryseobacterium*	0.93	0.04

Table S5. Bacterial indicator OTUs at day 28 based on *Bsal* infection for *L. vulgaris* and *T. cristatus*.

2.2 Figures

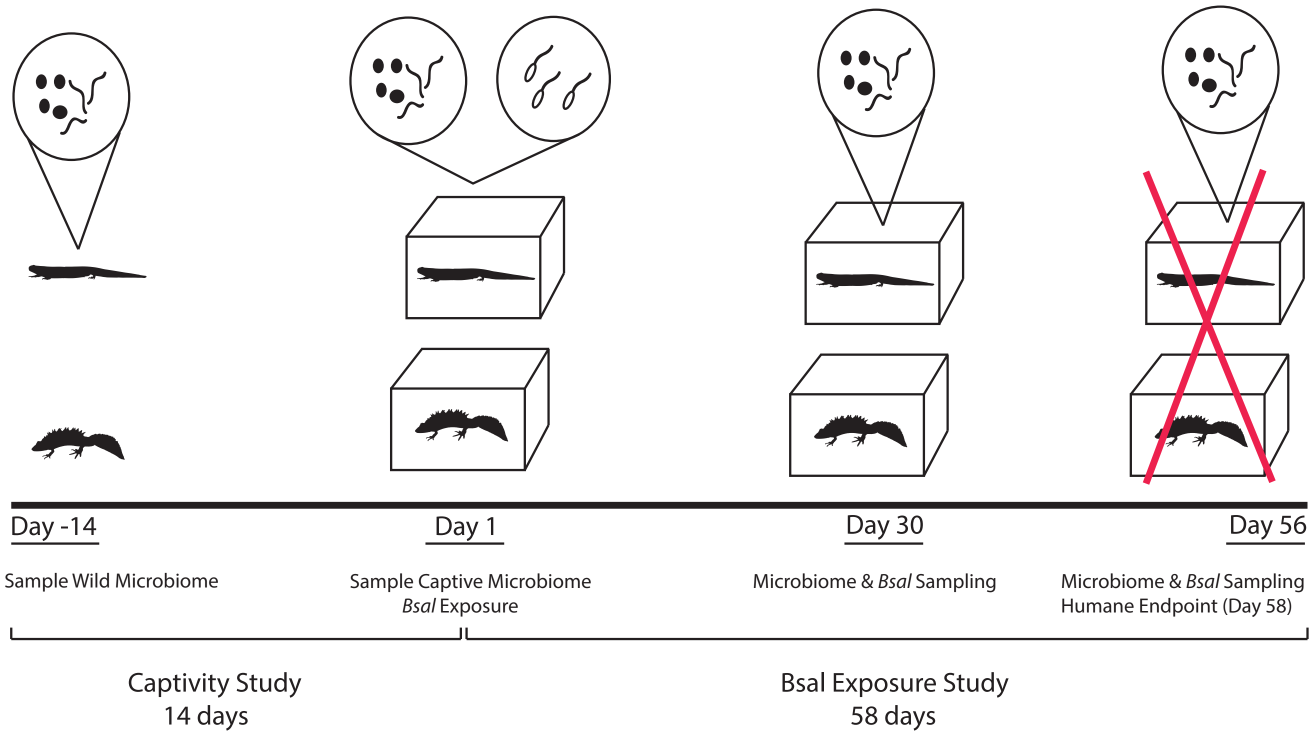

Figure S1. Overview of design for captivity study and *Bsal* exposure experiment.

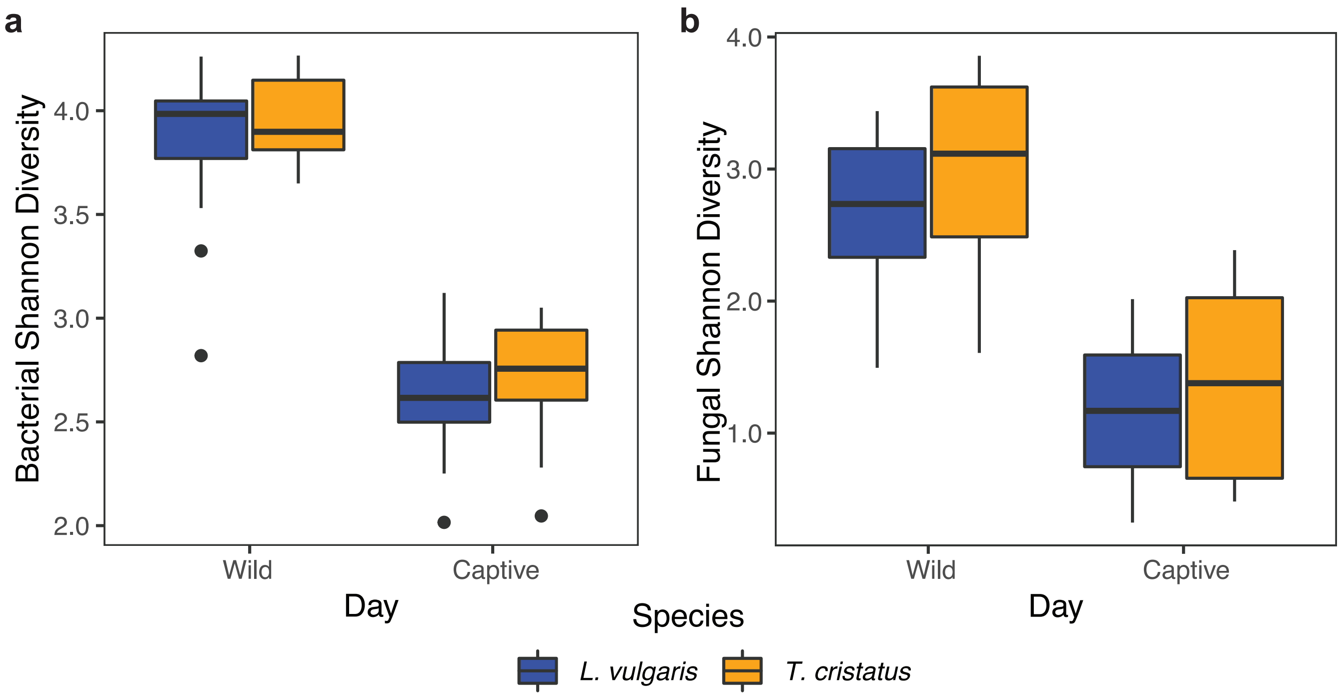

Figure S2. Skin Shannon diversity for (**A**) bacteria and (**B**) fungi in wild and captive *L. vulgaris* and *T. cristatus*.

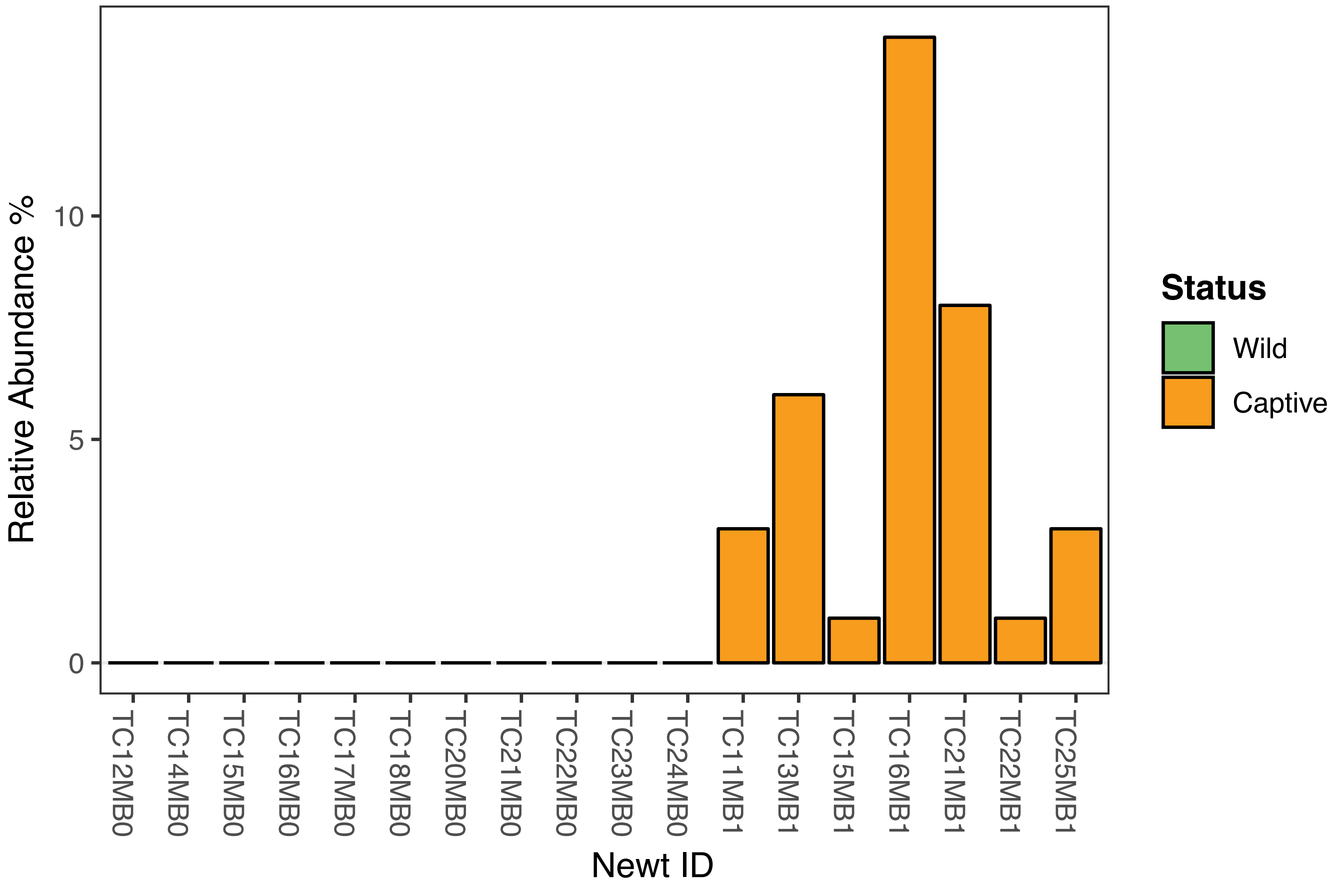

Figure S3. Plot of relative abundance of *Basidiobolus ranarum* on *T. cristatus* in the wild versus captivity.

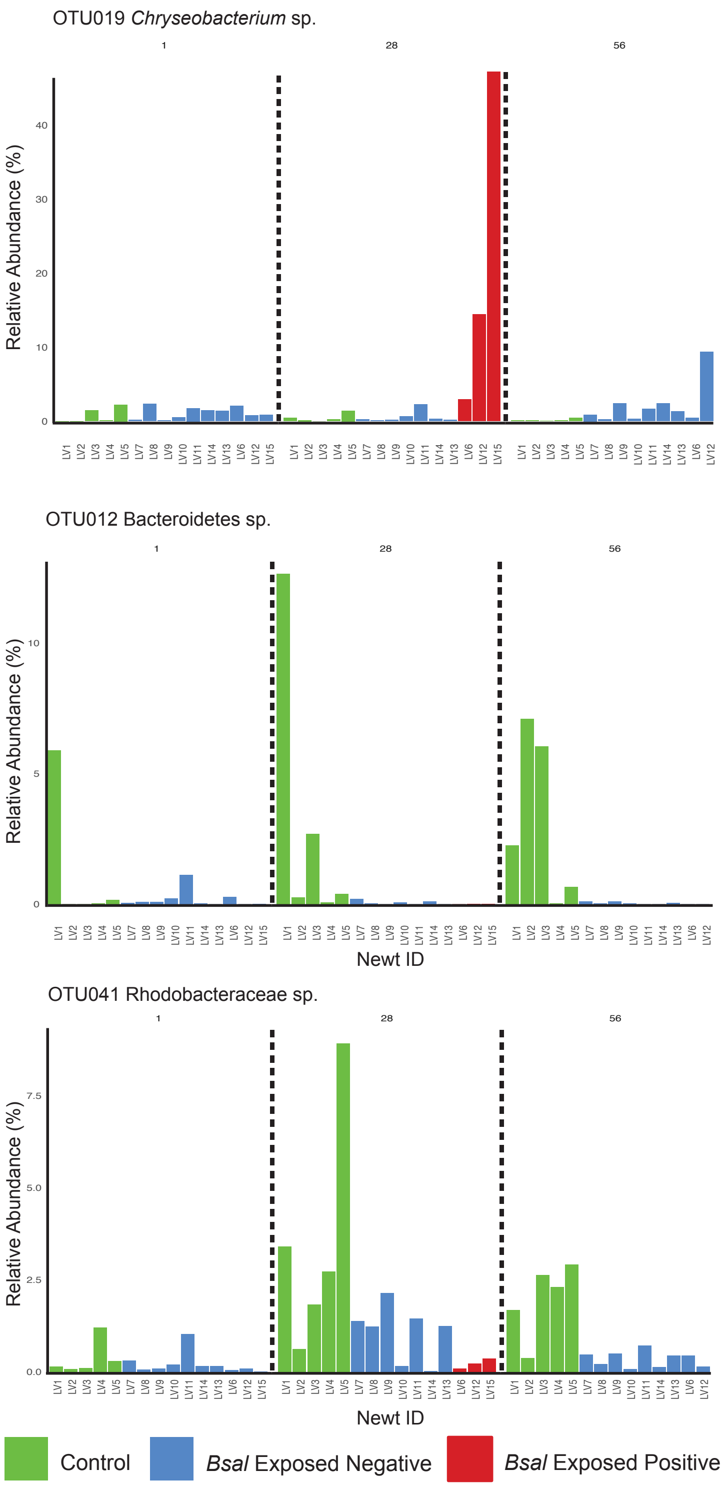

Figure S4. Plots of *L. vulgaris* indicator taxa relative abundance at days 1, 28 and 56 of the *Bsal* exposure experiment.
